# Hybrid Polymer–Inorganic Materials with Hyaluronic Acid as Controlled Antibiotic Release Systems

**DOI:** 10.3390/ma17010058

**Published:** 2023-12-22

**Authors:** Kamila Lis, Joanna Szechyńska, Dominika Träger, Julia Sadlik, Karina Niziołek, Dagmara Słota, Josef Jampilek, Agnieszka Sobczak-Kupiec

**Affiliations:** 1 Department of Materials Science, Faculty of Materials Engineering and Physics, Cracow University of Technology, 37 Jana Pawła II Av., 31-864 Krakow, Polandkarina.niziolek@doktorant.pk.edu.pl (K.N.);; 2 Jerzy Haber Institute of Catalysis and Surface Chemistry, Polish Academy of Sciences, Cracow, 8 Niezapominajek, 30-239 Krakow, Poland; 3 Department of Analytical Chemistry, Faculty of Natural Sciences, Comenius University, Ilkovicova 6, 842 15 Bratislava, Slovakia; 4 Department of Chemical Biology, Faculty of Science, Palacky University, Slechtitelu 27, 783 71 Olomouc, Czech Republic

**Keywords:** clindamycin, collagen, drug delivery system, hyaluronic acid, brushite, composites

## Abstract

In recent years, significant developments have taken place in scientific fields such as tissue and materials engineering, which allow for the development of new, intelligent biomaterials. An example of such biomaterials is drug delivery systems that release the active substance directly at the site where the therapeutic effect is required. In this research, polymeric materials and ceramic–polymer composites were developed as carriers for the antibiotic clindamycin. The preparation and characterization of biomaterials based on hyaluronic acid, collagen, and nano brushite obtained using the photocrosslinking technique under UV (ultraviolet) light are described. Physical and chemical analyses of the materials obtained were carried out using Fourier transform infrared spectroscopy (FT-IR) and optical microscopy. The sorption capacities were determined and subjected to in vitro incubation in simulated biological environments such as Ringer’s solution, simulated body fluid (SBF), phosphate-buffered saline (PBS), and distilled water. The antibiotic release rate was also measured. The study confirmed higher swelling capacity for materials with no addition of a ceramic phase, thus it can be concluded that brushite inhibits the penetration of the liquid medium into the interior of the samples, leading to faster absorption of the liquid medium. In addition, incubation tests confirmed preliminary biocompatibility. No drastic changes in pH values were observed, which suggests that the materials are stable under these conditions. The release rate of the antibiotic from the biomaterial into the incubation medium was determined using high-pressure liquid chromatography (HPLC). The concentration of the antibiotic in the incubation fluid increased steadily following a 14-day incubation in PBS, indicating continuous antibiotic release. Based on the results, it can be concluded that the developed polymeric material demonstrates potential for use as a carrier for the active substance.

## 1. Introduction

Over the past few years, more people have been struggling with chronic treatment associated with bone loss caused by disease or accidents. Researchers are using tissue engineering (TE) to regenerate bone tissue, developing new materials to mimic the natural properties of the tissue [1,2,3]. To achieve this, modern technologies are being sought for the design and development of biomaterials that play an important role in TE applications [4,5,6,7]. The main products that are used for tissue regeneration are hydrogels, which consist of a three-dimensional network and have very good elasticity and low friction [8,9,10]. In the case of bone injuries, the best option is to introduce a composite consisting preferably of natural polymers combined with ceramics, which will be synthetic bone substitutes [11,12,13,14]. Such grafts must not only help with bone regeneration but should also mimic natural bones in their structure and strength so that the implant is not rejected by the body [15,16,17,18,19]. For better bone regeneration, the inserted implants should additionally have a drug in them to facilitate regeneration or relieve pain after surgery. For this purpose, drug delivery systems (DDS) that involve releasing the drug locally to achieve a therapeutic effect are used. The following section will describe the material that was used in our study [20]. The natural biomaterials used for tissue regeneration include biodegradable polymers, hydrogels, and porous scaffolds. Natural materials consist of proteins such as collagen and polysaccharides (e.g., hyaluronic acid). The properties of biomaterials vary depending on the composition, source, and isolation method. These materials have also found applications in drug delivery and cell growth factors [2,8].

One of the better-known polysaccharides used in the manufacture of coatings is hyaluronic acid (HA), a linear glycosaminoglycan (HAs have a wide range of molecular weights, from 20,000 to several million Daltons, depending on the enzyme catalyzing its synthesis) found in the human body [21,22,23]. It is composed of D-glucuronic acid and N-acetylglucosamine disaccharide units. It is hydrophilic and its main function is to hydrate the body thanks to its hydroxyl group, which can easily bind water molecules to the chain through hydrogen bonds [24,25]. In addition, due to its polymeric structure, its side groups, including carboxyl or acetamide groups, can be sites for many chemical reactions [22,26,27,28]. It is synthesized by hyaluronate synthase in the plasma membrane and is then extruded into the extracellular matrix. Its main locations are the connective tissues of the dermis, the dental pulp matrix, and the synovial fluids. It plays an important biological and physiological role in the human body, e.g., it contributes to maintaining the mechanical integrity of the tissue, viscoelasticity, and hydration in the extracellular matrix [28,29]. In addition, its important intracellular functions are cell adhesion and wound healing [30,31]. Moreover, hyaluronic acid promotes wound healing and inhibits inflammation depending on the MW size, which provides broad applications in tissue engineering [32]. The most common application of HA is in biomedicine, as manufactured hydrogels with hyaluronic acid can combine with proteins or drugs to help regenerate tissues and facilitate drug delivery. It can also be used in orthopedics for implant coatings [31].

Collagen may play an important role in DDS and bone tissue regeneration. It is one of the most ubiquitous proteins in living organisms [33]. It is essential for life and almost 90% of type I, II, and III collagen are found in the human body. There are about 28 types in nature, differing in structure and organization. They can be divided into multiple groups, including fibril-forming, fiber-associated, network-forming, and anchor-forming collagens [33,34,35]. Type I collagen is most often found in tissues such as bones and tendons. Interestingly, this collagen type can improve the mechanical strength or remodel new bones through the mineralization of mature bones. In contrast, type II and III collagens are found in cartilage and soft tissues. Collagen can be extracted from most animal tissues. Bovine skin and tendons are used for medical devices [36] as they are homologous to human collagen. The structure of collagen consists of a triple helix, but each type of collagen differs in structure, function, and tissue location. Collagen is used in the food and cosmetic industries. Its main function is to accelerate wound healing, repair cartilage and bone, and repair peripheral nerves [29,37].

Not only do biomaterials have regenerative and healing-accelerating properties, they can also be used to deliver drugs. In our study, we used clindamycin, an antibiotic with bacteriostatic and—at high concentrations—bactericidal effects [38,39]. The mechanism of action of this antibiotic is to inhibit bacterial protein synthesis by binding to the large subunit of the bacterial ribosome. In addition, clindamycin has favorable pharmacokinetic parameters as it is very rapidly absorbed from the gastrointestinal tract. It is absorbed in the form of clindamycin chloride, which is the active form, and begins its action in the body [40,41,42]. This antibiotic is highly recommended for surgical prophylaxis, e.g., surgical site infections [27,43,44,45], although it can be used in many other areas of medicine, including:

- Lower respiratory tract infections, which are caused by the anaerobic bacteria *Staphylococcus aureus*.
- An adjunctive drug in the treatment of lung abscesses.
- Infections of the ear, throat, and sinuses spread by streptococci.
- Infections of the skin and soft tissues, and even necrotizing infections [37].

Biopolymers have many wonderful properties that enable our bodies to regenerate, but they are not very resistant to friction. Ceramics, which are distinguished by their high durability, can be used to ensure that the coating does not wear off quickly. Brushite (DCPD) is used for this purpose. It is a ceramic material from the hydroxyapatite family with admixtures of metal ions such as iron manganese, magnesium, zinc, and phosphate anions, which are partially replaced by hydroxide anions [46]. The hardness of this material on the Mosh scale is 1, which means that it is a material that can be easily ground [47]. Depending on the admixtures, it can have many colors, including white, brown, green, or grey. Calcium hydrogen phosphate is chemically resistant as it does not react with strong acids or bases. Due to its good properties, DCPD can be used in many scientific fields, including the manufacture of bone implants, as it is biocompatible and does not cause allergic reactions. Because it can be easily ground, it can be adapted to the shape and size of the defect [48,49,50,51]. It can also be used in the manufacture of creams and ointments, as it helps with treating acne and skin irritations. Additionally, it can be used as a dental filler. It is hard and can look very natural due to its white color [46,52].

## 2. Materials and Methods

### 2.1. Reagents

Reagents for the synthesis of brushite, i.e., disodium hydrogen phosphate (Na_2_HPO_4_·2H_2_O), hydrated calcium acetate (Ca(NO_3_)_2_·4H_2_O), and ammonia water (NH_4_OH, 25%) and reagents for the preparation of simulated body fluid (SBF) and Ringer’s fluid, i.e., NaCl, NaHCO_3_, KCl, K_2_HPO_4_·3H_2_O, MgCl_2_·6H_2_O, 1M HCl, CaCl_2_, Na_2_SO_4_, and Tris were purchased from Chempur (Piekary Śląskie, Poland). Phosphate-buffered saline (PBS) was prepared from Oxoid tablets (Basingstoke, UK). Polymer matrices were prepared using type II collagen (COL) (Yango, Warsaw, Poland). Hyaluronic acid (HA), (2-hydroxy-2-methylpropionate) was used as a photoinitiator, and poly(ethylene glycol) diacrylate (PEGDA) Mn 575 (Sigma-Aldrich, Darmstadt, Germany) was used as a crosslinking agent. The active substance was 98% clindamycin hydrochloride monohydrate from Abcr GmbH (Karlsruhe, Germany).

### 2.2. Preparation of Materials

#### 2.2.1. Preparation of Brushite

Ceramic phase brushite was obtained from disodium hydrogen phosphate (Na_2_HPO_4_·2H_2_O) and hydrated calcium acetate (Ca(NO_3_)_2_·4H_2_O) using the wet precipitation method. Briefly, 500 mL each of aqueous solutions of Na_2_HPO_4_∙2H_2_O and Ca(NO_3_)_2_∙4H_2_O were prepared, with each solution having a concentration of 0.5 mol/L. The Na_2_HPO_4_∙2H_2_O solution was placed on a magnetic stirrer and the prepared Ca(NO_3_)_2_∙4H_2_O solution was added at a rate of 1 drop per second. The pH was checked and maintained at 6–6.5 by adding 25% ammonia water. The resulting powder was aged for 24 h at room temperature, after which the precipitate was filtered and washed with distilled water to obtain a neutral pH. The product was then dried in a laboratory dryer for 4 h at 104 °C. This method of synthesizing brushite was described in a previous article [53].

#### 2.2.2. Preparation of Composites

To obtain polymeric materials, aqueous solutions of hyaluronic acid (1%) and collagen (15%) were prepared. Then, the appropriate amounts of acid and reagents were mixed based on the proportions in Table 1. A 2 mL volume of PEGDA—a crosslinking agent with a molecular weight of 575—and 50 µL of 2-hydroxy-2-methylpropionate—a photoinitiator—were added and the mixture was mixed thoroughly on an IKA model RCT ST magnetic stirrer (Königswinter, Germany). The entire solution was then transferred to a 10 cm diameter Petri dish and subjected to UV photocrosslinking using a C-type UV lamp from Medilux UV 436 HF (Medilux, Korntal-Münchingen, Germany) for 4 min (λ = 320 nm, 180 W) at room temperature. 

As the entire volume of the beaker in which the components were mixed was transferred to the petri dish, all the components were utilized to synthesize the materials; thus, the method generates no waste or by-products, as a hydrogel and/or composite solid is obtained directly from the liquid.

**Figure 1:**
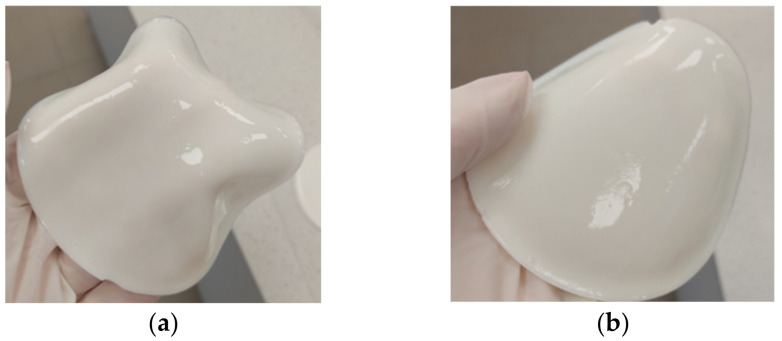
Flexible materials obtained via the photocrosslinking technique: (**a**) polymer material, sample no. 1 based on HA; (**b**) composite material, sample no. 1.1 with HA and DCPD.

Ceramic-phase composites were prepared in the same way by adding appropriate amounts of brushite (nanometric size) to the mixture. The polymeric materials and composites obtained by this method were characterized by high flexibility. Figure 1a illustrates an example of polymer material 1 and Figure 1b illustrates ceramic–polymer composite 1.1.

#### 2.2.3. Preparation of Drug Delivery Systems

The materials were modified using the active substance, clindamycin. This is an antibiotic used in the treatment of many conditions, including bacterial infection of bones and joints. To prepare the materials, the drug was dissolved in a 1% hyaluronic acid solution. This solution was used to synthesize the composites described in Section 2.2.1. The amount of clindamycin contained in the hyaluronic acid solution (10 mL) was 20 mg (i.e., 2 mg/mL). PEGDA 575 and the photoinitiator were added sequentially to 10 mL of hyaluronic acid containing the drug. Then, the mixture thus prepared was divided in half, and two polymeric materials with clindamycin were prepared; thus, the drug content in each carrier was 10 mg.

### 2.3. In Vitro Incubation in Biological Fluids 

#### 2.3.1. Electrochemical Analysis

The previously obtained samples were incubated in vitro in an artificial body fluid environment for 20 days. The biomaterials were immersed in 80 mL of simulated body fluids consisting of simulated body fluid (SBF), phosphate buffer saline (PBS), Ringer’s fluid, and distilled water, whose compositions are shown in Table 2. Incubation was performed at the temperature corresponding to human body temperature, which was precisely 36.6 °C. Electrochemical analysis was carried out during the incubation period. To monitor changes in pH value, potentiometric analysis was performed. The conductivity of the ions contained in the artificial body solutions was examined simultaneously. The purpose of the electrochemical analysis was to determine the occurrence of reactions between the composites and the fluids.

Electrochemical analysis was performed after 1, 8, 15, and 20 days of incubation. Measurements of pH changes and conductivity values were collected using an Elmetron CX-701 instrument (Zabrze, Poland). Table 2 shows the composition of the fluids.

#### 2.3.2. Determination of Sorption Capacity

The aim of the measurement is to test the sorption capacity of a material with a specific composition. The prepared samples are immersed in sterile and sealed containers, which are filled with PBS solution (60 mL) and then incubated at 36.6 °C for 48 h. The samples are then removed from the container, lightly drained of excess liquid on filter paper, and weighed. The sorption capacity is then calculated using Formula (1):

(1) $$Swellingcapacity=\frac{m_{1}-m_{0}}{m_{0}}\;\cdot 100\%$$

where

*m*_1_—the mass of the sample at the specified incubation time

*m*_0_—the dry mass of the sample.

### 2.4. Determination of Drug Release

The material modified by the antibiotic was incubated in 15 mL of PBS for 336 h (14 days). Then, using an MPW-260R centrifuge (Warsaw, Poland), the incubation liquid(1 mL) was centrifuged at 15,000 rpm at 4 °C. To investigate the amount of clindamycin hydrochloride released from the samples, the collected incubation fluids were analyzed using a high-performance liquid chromatography (HPLC) equipment from Shimadzu (Kyoto, Japan). The mobile phase was composed of acetonitrile (45%) and potassium dihydrogen phosphate (55%, pH 7.5). The measurement was carried out for 9 min using a detector set at 210 nm.

### 2.5. Morphology Analysis

To determine the morphology of the obtained materials, the surface morphology was analyzed using an optical microscope. This technique also allows you to detect defects and structural changes. The study was carried out on a KEYENCE digital microscope model VHX-7000 (Osaka, Japan). Analysis was performed on all samples, both polymers and samples with added DCPD, at room temperature.

### 2.6. Fourier-Transform Infrared Spectroscopy Analysis

Fourier Transform Infrared spectroscopy (FT-IR) was performed to identify the functional groups of the obtained composites with and without ceramic phase DCPD, as well as the pure components. For this purpose, a Thermo Scientific Nicolet iS5 FTIR spectrophotometer with an ATR (Attenuated Total Reflection) modelID7 (Thermo Scientific, Loughborough, UK) attachment was selected. The diamond crystal ATR in the instrument allows sufficient contact between the sample and the instrument, resulting in high-resolution test results. The register range of the spectra remained between 4000 cm^−1^ and 400 cm^−1^ at room temperature, with 32 scans at 4.0 cm^−1^ resolution.

## 3. Results

### 3.1. Incubation in Biological Fluids

#### 3.1.1. Electrochemical Analysis: Potentiometry

**Figure 2:**
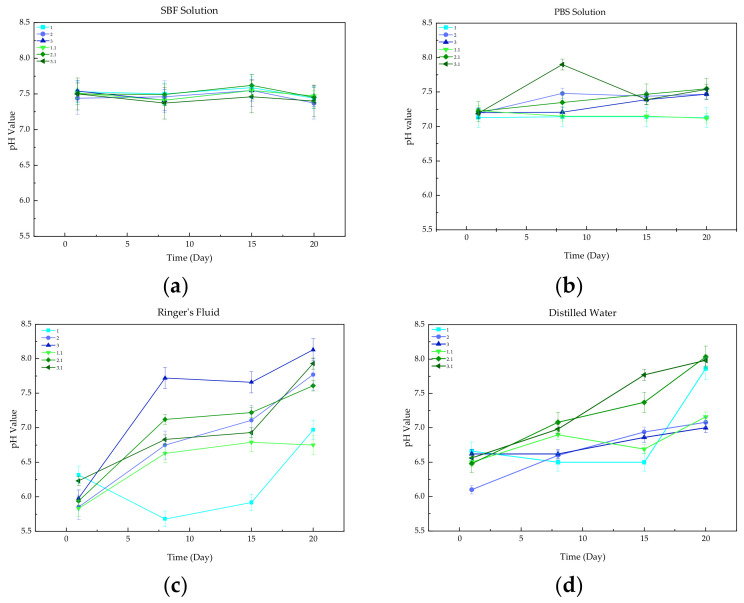
Measured pH values of (**a**) SBF solution, (**b**) PBS solution, (**c**) Ringer’s fluid, and (**d**) Distilled water.

A potentiometric study was performed to examine the effect of the obtained materials on the change in the pH of artificial body fluids during in vitro incubation. The biomaterials were immersed in four solutions: SBF, PBS, Ringer’s fluid, and distilled water. The resulting graphs of the changes in pH values throughout the incubation period are shown in Figure 2.

The largest changes in pH were observed for samples immersed in Ringer’s fluid. These observations were due to the degradation process, resulting in a reaction between ions released from the samples and ions originating from the solution. In addition, a reason for the significant increase in the pH values of the composites appeared to be the leaching of the calcium hydrogen phosphate. DCPD is a poorly soluble biomaterial due to its inorganic nature, which results in higher alkalinity of the solutions. In the case of distilled water, a minimal linear increase in pH value was observed due to the absence of buffering properties of the water. pH was most stable throughout the incubation of the PBS and SBF solutions, due to the formation of new apatite layers on the surfaces of the samples resulting in the slowing down of the degradation of biomaterials.

All the observed changes in pH caused by the presence of composites remained within a safe range for the human body.

#### 3.1.2. Electrochemical Analysis: Conductivity

**Figure 3:**
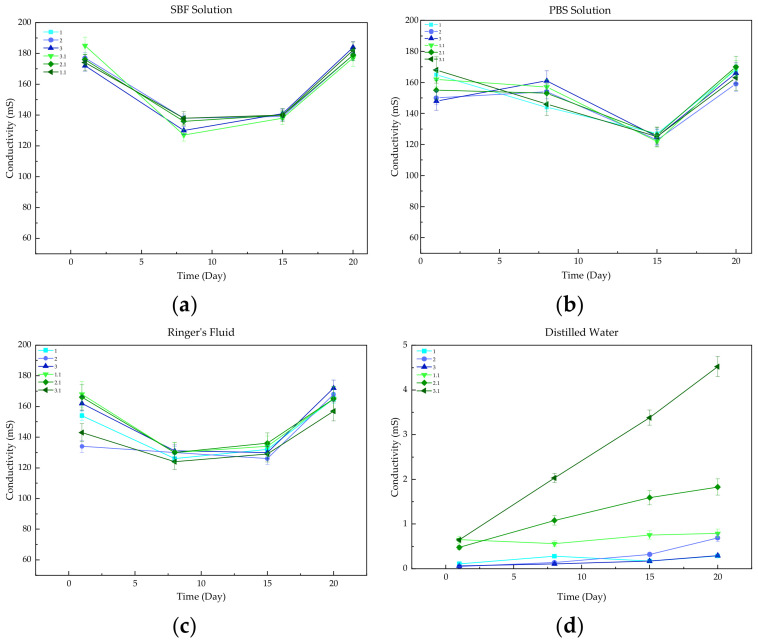
Measured conductivity parameters for (**a**) SBF solution, (**b**) PBS solution, (**c**) Ringer’s fluid, and (**d**) Distilled water.

The study of electrochemical conductivity was based on the change in ion concentration during in vitro incubation in an artificial environment of body fluids. The results of this examination, presented in the form of graphs of changes in the conductivity parameter during the incubation period, are shown in Figure 3. Changes in conductivity values were caused by ion exchange between the incubation fluids and the material. The changes became more noticeable as time passed.

There were minimal changes in the conductivity values of all the solutions, with no abrupt changes. A similar degree of conductivity was observed in both Ringer’s fluid and SBF solution, as shown by the associated curves. The changes in the conductivity parameter were similar for all samples in the PBS solution, as was the absence of burst changes in conductivity values. The high stability of the composites immersed in Ringer’s fluid, SBF, and PBS is due to the formation of apatite layers on the surface of each of the materials, which slows down the release of free ions and reduces the rapid increases in their concentrations. However, the samples immersed in distilled water showed the greatest stability throughout the incubation time. The reason for this behavior is the low concentration of free ions in the distilled water.

### 3.2. Determination of Sorption Capacity

The swelling ability of each sample was determined after incubation in PBS. This fluid was chosen because drug release determination was conducted in PBS.

**Figure 4:**
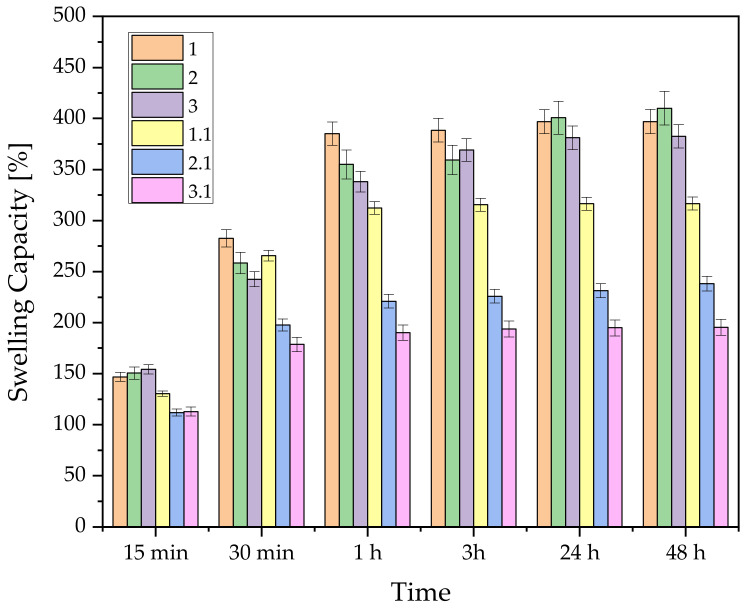
Determination of sorption capacities for composites consisting of natural and ceramic-modified polymers.

The swelling effect was confirmed in all materials, both polymeric and composites. From the analysis of the results (Figure 4), it was observed that the hydrogels with only biopolymers in their composition had the highest sorption capacity, which varied between 382% (for sample 3) and 410% (for sample 2). For the hydrogels modified with DCPD, the swelling capacity was between 195% for sample 3.1 and 317% for sample 1.1. The highest sorption capacity was observed for polymer samples 1 and 2, which had similar results after 48 h. Considering that in previous measurements it was the hydrogel based on hyaluronic acid that swelled the most, it is assumed that, after 48 h, an error occurred during the measurement of sample 2, and the excess fluid was not collected accurately. This theory would coincide with the results of the composite materials, as they show that samples with collagen have lower sorption capacities. Naturally, the reduction in sorption capacity in composites 1.1, 2.1, and 3.1 compared with their polymeric counterparts is associated with the presence of a ceramic phase between the polymer chains, as demonstrated in earlier reports [54,55].

### 3.3. Determination of Drug Release

**Figure 5:**
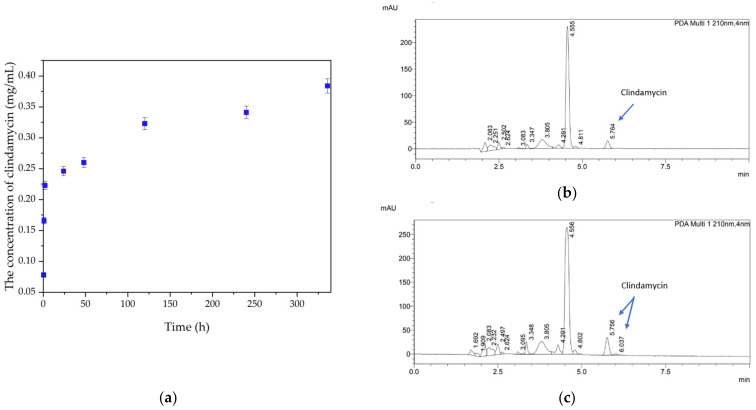
(**a**) Quantities of clindamycin (mg/mL) released by sample 1 over time. (**b**,**c**) Chromatograms of sample 1 incubated in PBS after 0.5 h (**b**) and 336 h (**c**).

The percentages of clindamycin released from composite samples are shown in Figure 5. As can be seen, after 14 days of immersion, clindamycin concentration in the incubation fluid was five times higher than its concentration after half an hour. Chromatograms of sample 1 showed peaks characteristic of clindamycin (retention time of about 5 and 7 min) and other peaks corresponding to other constituents of the matrix (Figure 5). Two characteristic peaks corresponding to the antibiotic appeared on the chromatogram after 14 days (5, 7, and 6 min). The results indicate that the release of clindamycin is time-dependent. Furthermore, it was shown that for sample 1, the clindamycin content in the fluid increased continuously during incubation, suggesting that although the samples did not swell further, the antibiotic was still released. This may be due to the initial release of clindamycin from the surface of the composite and the subsequent diffusion of the antibiotic from inside the structure.

### 3.4. Morphology Analysis

**Figure 6:**
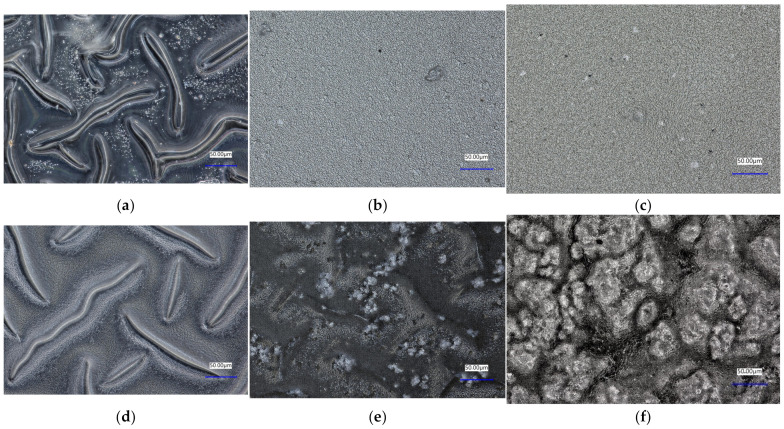
Analysis of the surface morphology of (**a**) polymer sample 1, (**b**) polymer sample 2, (**c**) polymer sample 3, (**d**) polymer sample 1 with drug, (**e**) composite sample 2.1 top, and (**f**) composite sample 2.1 bottom.

To determine the morphology of the obtained polymer materials and the ceramic–polymer composites, the surface morphology was analyzed using an optical microscope (Figure 6). Characteristic irregularities and indentations were observed in polymer material number 1. It should be noted that no characteristic indentations were observed for hyaluronic acid in sample number 2, meaning that collagen dominated over the effect of acid in the structure. Samples 2 and 3 had relatively similar structures. Images of the ceramic phase composites (Figure 6e,f) were taken from both sides (top and bottom), as the brushite sedimented during the crosslinking process. The observed structures of these composites differ significantly from side to side. From the bottom, spherical brushite particles can be seen forming agglomerates resembling a cauliflower structure. On the other hand, single clusters of this phase and a significant part of the polymer matrix can be seen from the top. In contrast, the clindamycin-modified sample resembles the structure of its material before modification. No change in the structure of sample 1 was observed after the addition of clindamycin. Microscopic images confirmed that the composite sample is not homogeneous, as agglomeration of brushite was observed on one side. However, this does not exclude the application potential of the sample, as gradient materials are often used as coating materials. 

### 3.5. Fourier-Transform Infrared Spectroscopy Analysis

**Figure 7:**
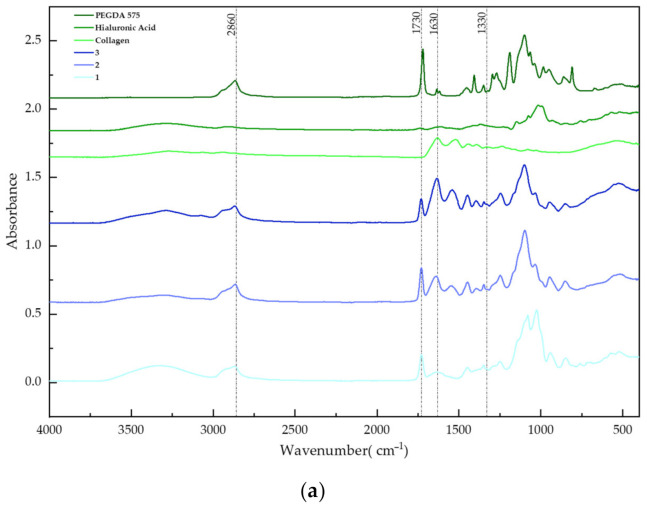

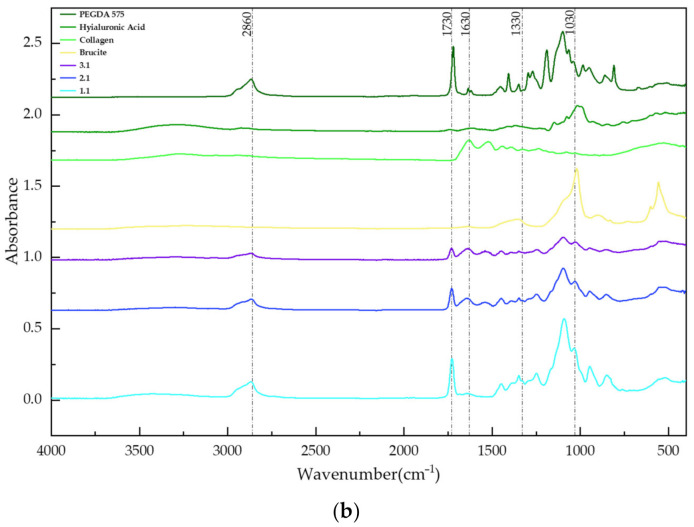
FT-IR spectra of pure components and samples of (**a**) polymer materials and (**b**) ceramic–polymer composites.

FT-IR spectra were used to analyze the chemical composition of the remaining materials and composites. The spectra of the pure components and the formed biomaterials, with and without DCPD, are shown in Figure 7.

In all of the composites, bands belonging to the crosslinking agent PEGDA 575 were distinguished at a wavelength of 2680 cm^−1^. In the biomaterials containing collagen, prominent bands corresponding to the functional group -CO-NH_2_ were observed at a wavelength of 1630 cm^−1^, originating from amide II, as well as less visible bands appearing at 1330 cm^−1^, originating from the vibrations of amide III. In samples containing hyaluronic acid, significant groups were present between 400–1700 cm^−1^. Phosphate groups were identified on the surface of the biomaterials containing calcium hydrogen phosphate, occurring at wavelengths between 550 cm^−1^ and 1030 cm^−1^.

## 4. Discussion

This study describes the synthesis of polymer materials and polymer–ceramic composites. Based on the tests performed, the samples obtained had fundamental physiochemical properties. In addition, the polymer composites demonstrated the potential of the active substance carrier.

FT-IR spectrometry analysis was used to compare the chemical compositions of the pure components with those of prepared composites. Characteristic peaks corresponding to functional groups were observed in the pure components as well as in the resulting samples. In the composites, a peak corresponding to the chemical composition of calcium hydrogen phosphate was distinguished at a wavelength of 1030 cm^−1^. During the analysis of the polymer materials, peaks originating from hyaluronic acid were identified in samples 1 and 2 at wavelengths ranging from 400 cm^−1^ to 1700 cm^−1^. In sample 3, a peak characteristic of the -CO-NH_2_ functional group was detected at a wavelength of 1630 cm^−1^.

To determine the behavior of the biomaterials under artificial body fluids conditions, the samples were incubated in vitro. Incubation was performed in the presence of the following solutions: SBF, PBS, Ringer’s fluid, and distilled water. Potentiometric analysis was performed to determine the effect of the immersed samples on the change in the pH value of each of the fluids. The results showed minimal changes in pH value within the safe range for the organism. PBS and SBF solutions were found to be the most stable fluids. The high stability was due to the buffering properties of both solutions and the formation of apatite layers on the surfaces of samples. Conductivity was investigated simultaneously. The results indicated the occurrence of a reaction between free ions from the fluids and the biomaterials. Minimal changes in the conductivity parameter were observed in all fluids, and there were no abrupt changes. This study also confirmed that the materials are not inert but instead interact with the incubation fluids. This is also evidenced by microscopic analysis, as differences in surface morphology were observed before and after the incubation period.

To identify samples for further modification with the active substance, the composites were subjected to a sorption capacity test to determine their swelling parameters. Sample 1 was found to have the highest swelling index and was therefore modified by introducing clindamycin into the polymeric phase.

The modified sample was subjected to HPLC to define the rate of drug release. The results demonstrated that more than half of the clindamycin was released from the biomaterial. Drug release was observed over 14 days. The reason for this behavior is the initial release of the antibiotic from the surface of the composites and its subsequent diffusion from the center of the structure. Unfortunately, a drug release study was not conducted using the composites because the nanometric DCPD leached out during incubation. This is particularly evident in the conductivity plot of distilled water, where the largest conductivity changes were observed in composite samples.

The synthesized biomaterials should be investigated further to determine their potential as drug carriers. One of the recommended tests is a cytotoxicity test, which should be carried out to identify possible negative effects on living tissue.

## 5. Conclusions

The proposed method of synthesis using UV light enables the preparation of fully crosslinked and continuous polymeric and composite biomaterials that demonstrate some potential for use as carriers of active substances. The developed synthesis method does not generate by-products and does not require the use of hazardous or toxic substances, making it ecologically and environmentally safe. Preliminary biocompatibility was confirmed by incubating with fluids simulating biological environments; no drastic changes in pH values were observed, suggesting that the materials are stable under these conditions. Their potential for use as antibiotic carriers was confirmed by conducting studies to determine the amount of drug released; such targeted therapies have advantages over conventional oral drug intake as they eliminate the phenomenon of systemic drug distribution. Further studies of cytocompatibility against cell lines are required, particularly against bone-forming cells whose proliferation should be stimulated by the addition of DCPD and the presence of collagen.

## Figures and Tables

**Table 1:** Composition of biomaterials.

Sample Symbol	HA (g)	COL (g)	DCPD (% *w*/*v*)
1	10	-	-
2	5	5	
3	-	10	
1.1	10	-	5
2.1	5	5	
3.1	-	10	

**Table 2:** Composition of simulated biological fluids.

Component (g/L)	SBF Solution	Ringer’s Fluid	PBS Solution
NaCl	8.035	8.600	8.000
NaHCO_3_	0.355		
KCl	0.225	0.300	0.200
K_2_HPO_4_∙3H_2_O	0.231		
MgCl_2_∙6H_2_O	0.311		
1M HCl	40 mL		
CaCl_2_	0.292	0.243	
Na_2_SO_4_	0.072		
Tris	6.118		
Na_2_HPO_4_			1.150
KH_2_PO_4_			0.200

## Data Availability

The data that support the findings of this study are contained within the article.

## References

[1] Radulescu D.E., Neacsu I.A., Grumezescu A.M., Andronescu E. (2022). Novel Trends into the Development of Natural Hydroxyapatite-Based Polymeric Composites for Bone Tissue Engineering. Polymers.

[2] Niemczyk-Soczynska B., Zaszczyńska A., Zabielski K., Sajkiewicz P. (2021). Hydrogel, Electrospun and Composite Materials for Bone/Cartilage and Neural Tissue Engineering. Materials.

[3] Zhou B., Jiang X., Zhou X., Tan W., Luo H., Lei S., Yang Y. (2023). GelMA-based bioactive hydrogel scaffolds with multiple bone defect repair functions: Therapeutic strategies and recent advances. Biomater. Res..

[4] Zhou J., Zhang Z., Joseph J., Zhang X., Ferdows B.E., Patel D.N., Chen W., Banfi G., Molinaro R., Cosco D. (2021). Biomaterials and nanomedicine for bone regeneration: Progress and future prospects. Exploration.

[5] Kim H., Hwangbo H., Koo Y.W., Kim G. (2020). Fabrication of mechanically reinforced gelatin/hydroxyapatite bio-composite scaffolds by core/shell nozzle printing for bone tissue engineering. Int. J. Mol. Sci..

[6] Lopes D., Martins-Cruz C., Oliveira M.B., Mano J.F. (2018). Bone physiology as inspiration for tissue regenerative therapies. Biomaterials.

[7] Wang W., Yeung K.W.K. (2017). Bone grafts and biomaterials substitutes for bone defect repair: A review. Bioact. Mater..

[8] Agarwal R., García A.J. (2015). Biomaterial strategies for engineering implants for enhanced osseointegration and bone repair. Adv. Drug Deliv. Rev..

[9] Chen F.-M., Jin Y., Chen F.-M., Jin Y. (2010). Periodontal Tissue Engineering and Regeneration: Current Approaches and Expanding Opportunities. Tissue Eng. Part B Rev..

[10] Holtzclaw D., Toscano N., Eisenlohr L., Callan D. (2008). The Safety of Bone Allografts Used in Dentistry: A Review. J. Am. Dent. Assoc..

[11] Florencio-Silva R., Sasso G.R.D.S., Sasso-Cerri E., Simões M.J., Cerri P.S. (2015). Biology of Bone Tissue: Structure, Function, and Factors That Influence Bone Cells. BioMed Res. Int..

[12] Wijesinghe W., Mantilaka M., Senarathna K.C., Herath H., Premachandra T., Ranasinghe C., Rajapakse R., Edirisinghe M., Mahalingam S., Bandara I. (2016). Preparation of bone-implants by coating hydroxyapatite nanoparticles on self-formed titanium dioxide thin-layers on titanium metal surfaces. Mater. Sci. Eng. C.

[13] Jeong J., Kim J.H., Shim J.H., Hwang N.S., Heo C.Y. (2019). Bioactive calcium phosphate materials and applications in bone regeneration. Biomater. Res..

[14] Gorodzha S., Douglas T.E.L., Samal S.K., Detsch R., Cholewa-Kowalska K., Braeckmans K., Boccaccini A.R., Skirtach A.G., Weinhardt V., Baumbach T. (2016). High-resolution synchrotron X-ray analysis of bioglass-enriched hydrogels. J. Biomed. Mater. Res.-Part A.

[15] Douglas T.E.L., Dziadek M., Gorodzha S., Lišková J., Brackman G., Vanhoorne V., Vervaet C., Balcaen L., del Rosario Florez Garcia M., Boccaccini A.R. (2018). Novel injectable gellan gum hydrogel composites incorporating Zn- and Sr-enriched bioactive glass microparticles: High-resolution X-ray microcomputed tomography, antibacterial and in vitro testing. J. Tissue Eng. Regen. Med..

[16] Ripamonti U., Crooks J., Khoali L., Roden L. (2009). The induction of bone formation by coral-derived calcium carbonate/hydroxyapatite constructs. Biomaterials.

[17] Wang Z., Jiang S., Zhao Y., Zeng M. (2019). Synthesis and characterization of hydroxyapatite nano-rods from oyster shell with exogenous surfactants. Mater. Sci. Eng. C.

[18] Bas M., Daglilar S., Kuskonmaz N., Kalkandelen C., Erdemir G., Kuruca S.E., Tulyaganov D., Yoshioka T., Gunduz O., Ficai D. (2020). Mechanical and biocompatibility properties of calcium phosphate bioceramics derived from salmon fish bone wastes. Int. J. Mol. Sci..

[19] Chuysinuan P., Nooeaid P., Thanyacharoen T., Techasakul S., Pavasant P., Kanjanamekanant K. (2021). Injectable eggshell-derived hydroxyapatite-incorporated fibroin-alginate composite hydrogel for bone tissue engineering. Int. J. Biol. Macromol..

[20] Li C., Wang J., Wang Y., Gao H., Wei G., Huang Y., Yu H., Gan Y., Wang Y., Mei L. (2019). Recent progress in drug delivery. Acta Pharm. Sin. B.

[21] Zhai P., Peng X., Li B., Liu Y., Sun H., Li X. (2020). The application of hyaluronic acid in bone regeneration. Int. J. Biol. Macromol..

[22] Hemshekhar M., Thushara R.M., Chandranayaka S., Sherman L.S., Kemparaju K., Girish K.S. (2016). Emerging Roles of Hyaluronic Acid Bioscaffolds in Tissue Engineering and Regenerative Medicine.

[23] Einhorn T.A., Gerstenfeld L.C. (2015). Fracture healing: Mechanisms and interventions. Nat. Rev. Rheumatol..

[24] Fraser J.R.E., Laurent T.C., Laurent U.B.G. (1997). Hyaluronan: Its nature, distribution, functions and turnover. J. Intern. Med..

[25] Fallacara A., Baldini E., Manfredini S., Vertuani S. (2018). Hyaluronic acid in the third millennium. Polymers.

[26] Dreiss C.A. (2020). Hydrogel design strategies for drug delivery. Curr. Opin. Colloid Interface Sci..

[27] Eivazzadeh-Keihan R., Noruzi E.B., Aliabadi H.A.M., Sheikhaleslami S., Akbarzadeh A.R., Hashemi S.M., Gorab M.G., Maleki A., Cohan R.A., Mahdavi M. (2022). Recent advances on biomedical applications of pectin-containing biomaterials. Int. J. Biol. Macromol..

[28] Dicker K.T., Gurski L.A., Pradhan-Bhatt S., Witt R.L., Farach-Carson M.C., Jia X. (2014). Hyaluronan: A simple polysaccharide with diverse biological functions. Acta Biomater..

[29] Toole B.P. (2004). Hyaluronan: From extracellular glue to pericellular cue. Nat. Rev. Cancer.

[30] Choi B., Kim S., Lin B., Wu B.M., Lee M. (2014). Cartilaginous extracellular matrix-modified chitosan hydrogels for cartilage tissue engineering. ACS Appl. Mater. Interfaces.

[31] Balakrishnan B., Banerjee R. (2011). Biopolymer-based hydrogels for cartilage tissue engineering. Chem. Rev..

[32] Zamboni F., Wong C.K., Collins M.N. (2023). Hyaluronic acid association with bacterial, fungal and viral infections: Can hyaluronic acid be used as an antimicrobial polymer for biomedical and pharmaceutical applications?. Bioact. Mater..

[33] Walimbe T., Panitch A. (2020). Best of both hydrogel worlds: Harnessing bioactivity and tunability by incorporating glycosaminoglycans in collagen hydrogels. Bioengineering.

[34] Guillén-Carvajal K., Valdez-Salas B., Beltrán-Partida E., Salomón-Carlos J., Cheng N. (2023). Chitosan, Gelatin, and Collagen Hydrogels for Bone Regeneration. Polymers.

[35] Nabavi M.H., Salehi M., Ehterami A., Bastami F., Semyari H., Tehranchi M., Nabavi M.A., Semyari H. (2020). A collagen-based hydrogel containing tacrolimus for bone tissue engineering. Drug Deliv. Transl. Res..

[36] Fan L., Ren Y., Emmert S., Vučković I., Stojanovic S., Najman S., Schnettler R., Barbeck M., Schenke-Layland K., Xiong X. (2023). The Use of Collagen-Based Materials in Bone Tissue Engineering. Int. J. Mol. Sci..

[37] Ucar B. (2021). Natural biomaterials in brain repair: A focus on collagen. Neurochem. Int..

[38] Korzeniewska-Rybicka I., Karpinska A. (2018). Klindamycyna—Kompletna monografia leku. Pediatr. Med. Rodz..

[39] Thadepalli H., Dhawan V.K. (1982). Clindamycin A Review of Fifteen Years of Experience. Rev. Infect. Dis..

[40] Pavlović N., Bogićević I.A., Zaklan D., Ðanić M., Goločorbin-Kon S., Al-Salami H., Mikov M. (2022). Influence of Bile Acids in Hydrogel Pharmaceutical Formulations on Dissolution Rate and Permeation of Clindamycin Hydrochloride. Gels.

[41] Han S.S., Ji S.M., Park M.J., Suneetha M., Uthappa U.T. (2022). Pectin Based Hydrogels for Drug Delivery Applications: A Mini Review. Gels.

[42] Smieja M. (1998). Current indications for the use of clindamycin: A critical review. Can. J. Infect. Dis..

[43] Rial-Hermida M.I., Rey-Rico A., Blanco-Fernandez B., Carballo-Pedrares N., Byrne E.M., Mano J.F. (2021). Recent Progress on Polysaccharide-Based Hydrogels for Controlled Delivery of Therapeutic Biomolecules. ACS Biomater. Sci. Eng..

[44] Manzoor A., Dar A.H., Pandey V.K., Shams R., Khan S., Panesar P.S., Kennedy J.F., Fayaz U., Khan S.A. (2022). Recent insights into polysaccharide-based hydrogels and their potential applications in food sector: A review. Int. J. Biol. Macromol..

[45] Assefa M. (2022). Inducible Clindamycin-Resistant Staphylococcus aureus Strains in Africa: A Systematic Review. Int. J. Microbiol..

[46] Aqib M., Anwar A., Ajaz H., Akbar S., Manzoor A., Abid M., Waheed Z., Kanwal Q. (2023). Metal-Doped Brushite Cement for Bone Regeneration. J. Bionic Eng..

[47] Yassine I., Joudi M., Hafdi H., Hatimi B., Mouldar J., Bensemlali M., Nasrellah H., Mahammedi M.A.E., Bakasse M. (2022). Synthesis of brushite from phophogypsum industrial waste. Biointerface Res. Appl. Chem..

[48] Moses J.C., Dey M., Devi K.B., Roy M., Nandi S.K., Mandal B.B. (2019). Synergistic Effects of Silicon/Zinc Doped Brushite and Silk Scaffolding in Augmenting the Osteogenic and Angiogenic Potential of Composite Biomimetic Bone Grafts. ACS Biomater. Sci. Eng..

[49] Boanini E., Silingardi F., Gazzano M., Bigi A. (2021). Synthesis and Hydrolysis of Brushite (DCPD): The Role of Ionic Substitution. Cryst. Growth Des..

[50] Hurle K., Oliveira J.M., Reis R.L., Pina S., Goetz-Neunhoeffer F. (2021). Ion-doped Brushite Cements for Bone Regeneration. Acta Biomater..

[51] Vahabzadeh S., Fleck S., Duvvuru M.K., Cummings H. (2019). Effects of Cobalt on Physical and Mechanical Properties and In Vitro Degradation Behavior of Brushite Cement. JOM.

[52] Bohner M., Gbureck U., Barralet J.E. (2005). Technological issues for the development of more efficient calcium phosphate bone cements: A critical assessment. Biomaterials.

[53] Słota D., Piętak K., Florkiewicz W., Jampílek J., Tomala A., Urbaniak M.M., Tomaszewska A., Rudnicka K., Sobczak-Kupiec A. (2023). Clindamycin-Loaded Nanosized Calcium Phosphates Powders as a Carrier of Active Substances. Nanomaterials.

[54] Słota D., Florkiewicz W., Piętak K., Pluta K., Sadlik J., Miernik K., Sobczak-Kupiec A. (2022). Preparation of PVP and betaine biomaterials enriched with hydroxyapatite and its evaluation as a drug carrier for controlled release of clindamycin. Ceram. Int..

[55] Słota D., Florkiewicz W., Piętak K., Szwed A., Włodarczyk M., Siwińska M., Rudnicka K., Sobczak-Kupiec A. (2021). Preparation, Characterization, and Biocompatibility Assessment of Polymer-Ceramic Composites Loaded with Salvia officinalis Extract. Materials.

